# 

**Published:** 1910-10-15

**Authors:** 


					THE FUTURE t?? HOSPITALS
MEDICAL RELIEF FOR ALL ACCORDING TO MEANS.
A Scheme of Co-operation between the PATIENTS, the HOSPITALS,
and the STATE.
Price 2d. By Sir HENRY BURDETT, K.C.B., K.C.V.O. Price 2d.
THE SCIENTIFIC PRESS, Ltd., 28 & 29 Southampton St., Strand, London, W.C.,
and at all Bookstalls.
October 15, 1910. THE HOSPITAL
Publications.
By Sir WILLIAM BENNETT, K.C.V.O., F.R.C.S,
RECENTLY PUBLISHED. Crown 8vo. 5s. net.
INJURIES AND DISEASES OF
THE KNEE JOINT.
With 34 Illustrations.
iTISBET &; CO.
Also, 8vo. 6S. FOURTH EDITION.
MASSAGE AND MOVEMENTS IN
RECENT FRACTURES;
Sprains and their Consequences; Rigidity of
the Spine and the Management of Stiff Joints.
With. 23 Illustrations.
:n o 3sr <3- uvn .a. nsr s , gbeen c o.
Just Issued. Ninth Edition, Rewritten and Enlarged, 9s. net.
PHARMACY, MATERIA MEDIGA & THERAPEUTICS.
Being a Commentary on the B.P. and its Drugs, with their Preparations,
and on all the new Non-official Remedies in use, with Chapters on the
different processes used in Dispensing, Prescription-writing, Latin
Glossary, &c. By Sip W. WHITLA, M.D., LL.D., Professor, Materia
Medica, Queen's University, Belfast, and Senior Physician, Royal Victoria
Hospital.
By the same Author. New Work. 1,900 pages. Crown8vo.25s.net.
PRACTICE OF MEDICINE.
Containing in two Handy Volumes a complete and independent Article on
the Etiology, Symptoms, and Diagnosis of every Medical Disease; arranged
in Dictionary Form for Practitioners and Students.
London: BailliiSre, Tixdall & Cox, 8 Henrietta Street, W.C.
Now Beady. Second Edition, Revised and Enlarged. Price 3s. 6d. net.
INSANITY IN EVERYDAY PRACTICE.
By E. G. YOUNGER, M.D., M.R.C.P., D.P.H.
London : Baillieue, Tixdai.l & Cox, 8 Henrietta Street, Covent Garden.
A MANUAL OF
MEDICAL EXERCISES.
By PERCY LEWIS, M.D., M.R.C.S.,
Hon. Medical Officer to Victoria Hospital, Folkestone.
66 pages, with 35 Illustrations, cloth, 1s. 6d. net; post free, Is. 7d.
H. K. LEWIS, 136 GOWER STREET, LONDON, W.C.
By A. H. TUBBY, M.S.
DEFORMITIES*
Six hundred pages, 303 Illustrations. Price 17s.
"Standard work on the subject in the English language."
British Medical Journal.
SURGERY OF PARALYSES.
By A. H. TUBBY, M.S.Lond., F.R.C.S.Eng., and
ROBERT JONES, F.R.C.S.E.
Illustrated by 93 figures and 68 cases. Pp. 301. 10s. net.
" Can confidently recommend it as a practical summary of the most
modern views."?Edinburgh Medical Journal.
" Indicates original and successful methods of treatment."
American Journal of Orthopaedic Surgery.
London : Macmillan & Oo., Ltd. New York : The Macmillan Co.
FIFTH EDITION. With 50 Illustrations. 5/- net.
THE SCHOTT METHODS OF THE TREATMENT
OF CHRONIC DISEASES OF THE HEART.
With an Account of the Nauheim Baths and of their Therapeutic Exercise
By W. BEZLY THORNE, M.E., M.R.O.P.
London : J. & A. Churchill, 7 Great Marlborough Street.
THERAPEUTICS OF THE CIRCULATION
By Sir LAUDER BRUNTON, Bart., M.D, D Sc., F.R.C.P., &c.,
Consulting Physician to St. Bartholomew's Hospital.
Published under the Auspices op the University op London.
With numerous Diagrams. Medium 8vo. 7s. 6d. net.
JOHN MURRAY, Albemarle Street, W.
APPOINTMENTS AND RESIGNATIONS.
Mr. Arthur J. Evans, F.R.C.S. Edin., has been
appointed visiting surgeon to the Liverpool Workhouse
Hospital, Brownlow Hill.
Mr. John Turner, the senior assistant medical super-
intendent of the Ersex County Asylum at Brentwood, has
been appointed to succeed Mr. G. Amsden, who will retire
at the end of this year.
The following appointments have been made at the
Dreadnought Hospital, Greenwich :?Mr. A. J. Walton,
M.S., F.R.C.S., M.B., B.Sc. Lond., of the London Hos-
pital, to be assistant surgeon; Mr. Norman S. Gilchrist,
M.B., Ch.B., Aberdeen, to be house physician; and Mr.
Victor Field Usher, M.B., Ch.B. Edin., and Mr. H.
Lewis Barker, M.B., B.S., Lond., M.R.C.S., L.R.C.P., to
be house surgeons.
Obituary.
The death is announced of Dr. Frederick William Dyce
Fraser, of London, who has died suddenly at Borthwick-
brae, Roxburghshire, at the age of fifty-five. Dr. Fraserr
whose father was Emeritus Professor Campbell Fraser, was
educated at Prague and Edinburgh, and graduated M.D?
in 1883 at the Scottish University, being commended
for his thesis. He became a Fellow of the Royal College
of Physicians of Edinburgh in the same year. His former
appointments included that of Professor of Anatomy and
Physiology at the Imperial University of Osaka, Japan, and
in the skin department at Charing Cross Hospital, and ire
the Teddington and Hampton Hospital he had also held
various posts.
Dr. John Anderson, C.I.E., has died in his seventieth,
year. He studied at the Royal Infirmary, Manchester,
and St. Andrews University, where he graduated M.D. in
1885. He entered the Army Medical Service in 1864,.
and served in India, being medical officer of " The Chest-
nut Troop," Royal Horse Artillery. He was selected by
the late Lord Ripon for accompanying him to India, and
returned with him on the termination of his Viceroyalty,
having been previously decorated. He retired from the
Army in 1885 with the rank of brigade surgeon, and
became physician to the "Dreadnought" Hospital for
Seamen and to King Edward's Hospital for Officers. He
was also appointed lecturer on tropical diseases at St-
Mary's Hospital. He became M.R.C.S. in 1861, L.S.A-
in 1862, and F.R.C.P. in 1896.
The death on October 5 is announced of Mr. Cecil H?
Leaf, M.B., F.R.C.S., surgeon to the Cancer and Gordon-
Hospitals, London. The deceased surgeon was educated
at Trinity College, Cambridge, and the London Hospital,,
where he held the usual resident appointments. He gradu-
ated at Cambridge in 1885, obtaining the M.A., M.B., and'
B.C. degrees, and was a member of the Senate. He studied
at the London Hospital, where he was a demonstrator of
anatomy, house surgeon, house physician, and clinical assis-
tant, and he became F.R.C.S. in 1895. In 1900 Mr. Leaf
was appointed assistant surgeon to the Cancer Hospital,
afterwards becoming one of the senior surgeons there. As.
the outcome of his first three years' work at the hospital he
published an account of the clinical causes of cancer of the-
breast and its prevention, showing that this particular form'
appeared five times more frequently in the married than in.1
the unmarried. Mr. Leaf was the author of several sur-
gical works, and was joint author of the Lancet experiments-
with chloroform in 1893. Although he had been aware for
over a year that his days were numbered, he remained at"
work until mid-August, when his health finally broke down.
Well known as a careful anatomist and a most painstaking
student of everything appertaining to cancer research,
Mr. Leaf shone more in the academical side of surgical
work than as an operator. On the subject of lymphatics-
and lymphatic glands he was a perfect mine of informa-
tion, and besides translating a French work on this subject
was also the author of a treatise on the surgical anatomy
of the lymphatics. Only forty-six at the time of his death,
his work on these subjects had rendered his name familiar
on the Continent as well as in this country. This lamented1
death is the second which has occurred this year among,
the senior staff of the Cancer Hospital; indeed, the gap'
due to Mr. Keyser's loss has not yet been filled up. Mr.
Leaf was surgeon-captain to* a Volunteer Engineer corps;
in London.
Radium in Australia.?Several months ago a settler in
the Northern Territory drew attention to the fact that
ordinary white glass there exposed to the full rays of the
sun for several weeks took on the colour of amethyst; there-
fore radium was about! Two months ago a miner dis-
covered a radio-active mineral at Wodgina, in the Pilbara
district, Western Australia. This has since been christened
pilbarite. The Government mineralogist reports upon a
2-lb. sample of pilbarite that it assays 31 per cent, of
thorium and 27 per cent, of uranium, the latter containing
71.2 centigrams per ton. He estimates the value .of pilbarite
at ?370 per ton. The ore is in nodules from a pea to a
walnut in size, the colour bright yellow, and the specific
gravity 4.7. Later we hear that a company has been formed
to work a mine a few miles from Broken Hill?a mine whose
copper ore is said to carry uranium and radium. There is a
Radium Hill Company at Sydney which is testing large
deposits of radio-active ores that occur at Olary, South
Australia. Thirty tons of this ore have been distributed
among English and Continental firms interested in the
matter, and a small parcel was submitted to the Director
of the Bairnsdale School of Mines (Victoria). His report
was made public recently, and was so satisfactory that
the Board decided to have fifteen tons tested by him on
commercial lines. The South Australian deposits of the
ore are said to be of low grade but very extensive.
Typewriting.?Literary, scientific, and other MSS.
neatly and expeditiously typewritten by Mrs. L. Mon-
chablon, 14 Marquess Road, Canonbury, N. We have
pleasure in recommending this lady's work, which will be
found thoroughly satisfactory in all respects.
Gifts and Bequests.
The Committee for the Removal of King's College Hos-
pital to South London has received a cheque for ?500 from
Lord Iveagh.
Mr. W. B. Gibbins, a member of the Society of Friends,
has announced that he will give ?1,000 towards the cost
of certain additions to the Stratford-on-Avon Hospital.
Miss Sarah Crampton, of Hale Barns, Cheshire, has
bequeathed one-eighth of her residuary estate to the
governors of the Altrincham Provident Dispensary and
Hospital.
Sir David Salomons has presented to the Tunbridge
Wells General Hospital an installation of the Rontgen Rays
apparatus and Finsen lamps. He has given also a valuable
piece of radium, to remain in the custody of the hospital,
but to be available for private practitioners on payment
of a small fee.
The treasurer of the Royal Halifax Infirmary has
received from an anonymous donor ?1,000 to endow an
adult bed. At a recent meeting of the Board of Manage-
ment ?1,000 was fixed as the sum for the endowment
of a' bed or cot in any of the wards, and the gift just
received is the first for the special endowment of an adult
bed. Seven cots in the children's ward, however, have
previously been endowed in a similar manner. This gift
. is entirely apart from the other anonymous donation of
? ?1,000 announced a few days ago for the fund to com-
memorate the reign of King Edward VII.
Within a week the fund to provide a Welsh national
memorial to King Edward has reached the splendid sum
of ?150,000. The idea is that a great national campaign
be undertaken against tuberculosis by three means?
(1) education, (2) dispensaries, and (3) sanatoria. There
have been some very generous gifts, notably two anonymous (
donations each of ?50,000. Mr. David Davies, M.P., hae
given ?25,000, and other large subscribers are Mr. W. J.
Thomas, Ynyshir, ?5,000; Miss Thomas, Llwynmadoc,
?5,000; Sir Alfred Mond, M.P., ?5,000; Mr. and Mrs.
W. E. G. Curre', Chepstow, ?2,000; Mies Talbot, Margam
Abbey (first donation), ?1,000; and Mr. William Jenkins,
Ocean Colliery Company, ?1,000. Mr. David Davies, M.P.,
is treasurer of the national fund. The Western Mail
(Cardiff) has opened an auxiliary fund, and in the first
forty-eight hours received subscriptions amounting to
?10,000.
Jottings.
TO-DAY IS HOSPITAL SATURDAY !
Grapes weighing 160 lb. have been gathered from the
vine at the Royal Hospital for Incurables, Putney Heath,,
and distributed among the 230 in-patients of the institu-
tion.
On Wednesday, October 19, Mrs. Harcourt will be
At Home at 4 p.m. at Nuneham Park, Oxford, to members
and associates of the Oxfordshire district of the League of
Mercy.
The Hon. Walter Rothschild will preside af the festival
dinner, postponed in consequence of the death of the
late King, of the City of London Hospital for Diseases,
of the Chest, to be held at the Trocadero Restaurant on
Thursday, November 10, 1910, at 6.30 for 7 o'clock p.m.
The Committee hope that they may have the honour of
the company of those gentlemen who accepted the former
invitation besides those to whom the former date was
inconvenient.
The proposal to erect a cottage hospital at Haywards
Heath as a Mid-Sussex district memorial to the late King
has been adopted. The cost is expected to be about
?3,000, towards which ?1,002 has been contributed, the
sum already promised being ?2,207, including the value
of a site given by Mr. J. Bradford and Captain C.
Sergison on the west side of Haywards Heath. Five
trustees?Mr. A. Spicer, Mr. W. Stevens, Mr. W. A.
Sturdy, Captain Sergison, and Mr. J. Bradford?have
been appointed.
Sir Savile B. Crossley, Bart., presided over the recent
very satisfactory quarterly meeting of the Board of
Delegates of the Hospital Saturday Fund. The Secretary
(Mr. A. W. Davies) reported that the receipts from
January to September had reached ?15,634, a total, so
far, over ?2,300 in advance of last year at the correspond-
ing period. During the quarter 744 applications were con-
sidered by the Dental Sub-Committee, who made grants to
644 applicants. Of the remaining cases 79 were adjudged
to pay the full cost. During the same period 14,019 " Let-
ters of Recommendation" to the various medical charities
were issued upon the application of the collectors. This
number included Convalescent Home and Sanatoria
Letters, of which 438 were on the part-payment scheme.
The attendances of patients at the Surgical Appliance
department numbered 2,757, and the payments made by
them amounted to ?359 2s. 5d. A new Committee of the
Fund has been formed for the borough of Kensington,
with Mr. D. Kennedy as hon. secretary. Arrangements for
the Annual Special Collection?to-day, October 15?have
been completed, and the local committees have all had
boxes, bills, etc., distributed. A number of suggested
alterations to the regulations of the local committees, intro-
duced by the Collection Committee, have been referred to
the Council.
TO HOSPITAL SECRETARIES AND OTHERS.
We invite contributions from any of our readers, and
Bhall be glad to pay, according to length, for items of newe
for the institutional section (including appointments, resig-
nations, gifts, etc.) which we may publish. All payments
for manuscripts used are made as early as possible after the
beginning of each quarter.
THE HOSPITAL October 15, 1910.
Name
Address
This Coupon must accompany manuscript or contributions-in-
tended for The Hospital.
The Medical Library.
Post Free.
" Photomicrography." A Practical Guide and In-
troduction. 41 Reproductions of original speci-
mens. 63 drawings illustrating instruments and
methods. By Edmund Spitta, M.R.C.S.,
F.R.A.S.    12s. Od.
" Clinical Diagnosis." A Practical Illustrated
Handbook of Chemical and Microscopic
Methods. By W. G. Aitchison Robertson, M.D.,
F.R.C.P. ...       3s. &d.
" Notes on General Practice." Danger Signs and
Common Pitfalls. By S. M. Hebblethwaite,
M.D     3s. 9d.
" Medical History from the Earliest Times." By E.
Withington, M.A., M.B. Oxon  12s. 6d.
Of all booksellers or of The Scientific Press, Limited,
28 and 29 Southampton Street, Strand, London, W.C.
GENERAL PRACTITIONERS'
CONTRIBUTIONS.
The Hospital devotes a special page to General
Practitioners' contributions. We therefore invite
from practitioners contributions based upon their
experience in the management of cases, and in the
treatment and diagnosis of disease; especially shall
we be prepared to welcome articles dealing, practi-
cally, with treatment, and with the use and value
of new remedies and methods.
No article should exceed 1,100 words, and, if
accepted, one guinea for a contribution of this
length will be paid to the writer after publication.
Each communication should be accompanied by a
stamped directed envelope for the return of the MS.
if found unsuitable.
Contributions should be written, or preferably
typed, on one side of the paper only, and all articles
sent in are accepted upon the distinct understanding
that they are forwarded to The Hospital only.
Correspondence.
Correspondence on all subjects is invited, but no
communication can be entertained if the name and
address of the correspondent are not given as a
guarantee of good faith, but not necessarily for pub-
lication. All correspondents should write on one
side of the paper only.
BUSINESS NOTICES.
Annual Subscriptions.
The rate of annual subscriptions to The
Hospital, either direct from the Office or through
agents, is: ?
For the United Kingdom  15s. a year.
For the Colonies and Abroad ... 17s. ,,
For the United Kingdom (with
Insurance Policy)  ?1 ,,
inclusive of postage in each case.
Letters relating to the Publishing, Sale and
Advertisement Departments must be addressed to
the Manager (not to the Editor): ?
The Manager,
The Hospital Building,
28 and 29 Southampton Street,
Strand, London, W.C.
THE IDEAL WORK OF REFERENCE.
BURDETT'S HOSPITALS & CHARITIES
1910 E DIT Km.
?be H?eatv:fBook of philanthropy ant> Ibospital annual
Edited by SIR HENRY BURDETT, K.C.B., K.C.V.O.
A Complete Guide and Directory of the Hospitals, Asylums, and Charitable Institutions
throughout the English-speaking World.
Contains Special New Chapters on :?
Voluntary Hospitals in 1909.?Soundness of the Voluntary System.?The need of greater zeal on the
part of those who accept office.?State-aided Hospitals in the U.S.A. and Canada.?The Appropriation
of Public Funds for the partial Support of Voluntary Hospitals in U.S.A. and Canada.?The Need of an
Apostle and of greater interest on the part of the Clergy, &c., &c.
TWENTY-FIRST YEAR OF PUBLICATION.
Over 1,000 pages. Price 10/6 net; Post Free, 10/11-
ORDER your copy of the Medical Whitaker NOW.
Publishers :
THE SCIENTIFIC PEESS, LIMITED, 28 & 29 Southampton Street, Strand, London, W.C.
TWO USEFUL HANDBOOKS FOR
SECRETARIES.
HOSPITAL EXPENDITURE: THE COMMIS=
SARIAT. For Hospital Matrons and Managers.
2/6, post free.
" This should prove a valuable handbook to those upon whom rests
the responsibility of hospital finance, and should be of interest to the
public as giving a fair idea of what this department should and does
cost."?Manchester Courier.
A LEGAL HANDBOOK FOR THE USE OF
HOSPITAL AUTHORITIES. By Leonard
Syer Bristowe, M.A. Oxon., Barrister-at-Law.
2/6, post free.
THE SCIENTIFIC PRESS, Ltd.
28/29 SOUTHAMPTON STREET, STRAND, LONDON, W.C.
Printed by SPOTTISWOODE & CO. Ltd., New-street Square, E.O.; and Published by the Proprietors, THE SCIENTIFIC PRESS, LIMITED,
at 28 & 29 Southampton Street, Stra?d, London, W.C.
To be had of all Booksellers and Newsagents in the United Kingdom, and at all the Railway Bookstalls of Messrs. W. H. Smith & Son.

				

